# Expression of mutant p53, c-erbB-2 and the epidermal growth factor receptor in transitional cell carcinoma of the human urinary bladder.

**DOI:** 10.1038/bjc.1991.211

**Published:** 1991-06

**Authors:** C. Wright, K. Mellon, P. Johnston, D. P. Lane, A. L. Harris, C. H. Horne, D. E. Neal

**Affiliations:** University Department of Pathology, Newcastle-upon-Tyne, UK.

## Abstract

Expression of the p53, the epidermal growth factor receptor (EGFr; c-erbB-1) and c-erbB-2 proteins was studied in 82 patients with primary transitional cell carcinoma of the bladder using an immuno-histochemical method. Strong or moderate staining was found in 18% of tumours for p53 with weaker staining in a further 36% giving a total of 54% of tumours stained for p53. Strong staining was found in 15% of tumours for c-erbB-2 and in 31% for the EGFr. Tumours invading the bladder muscle were significantly more likely to be strongly stained positively for p53 and/or EGFr compared with superficial tumours: only 15% of invasive tumours were stained negatively for both p53 and EGFr. No statistical association was found between p53 and EGFr expression. Weakly positive associations were found between the expression of c-erbB-2 and p53 and between muscle invasive tumours and increased expression of c-erbB-2. Alterations in the expression of p53, c-erbB-1 and c-erbB-2 were found frequently in human transitional cell carcinoma of the urinary bladder and may be of clinical use in defining patient sub-groups of differing prognosis.


Br. J. Cancer (1991), 63, 967-970 © Macmillan Press Ltd., 1991

### Expression of mutant p53, c-*erb*B-2 and the epidermal growth factor receptor in transitional cell carcinoma of the human urinary bladder

C. Wright[1], K. Mellon[2], P. Johnston[1], D.P. Lane[3], A.L. Harris[4], C.H.W. Horne[1] & D.E. Neal[2]

*University Department of [1]Pathology and [2]Surgery/Urology, Newcastle-upon-Tyne; [3]ICRF Molecular Immunochemistry Laboratory, Potter's Bar, Herts.; and [4]Institute of Molecular Medicine, John Radcliffe Hospital, Oxford, UK.*

**Summary** Expression of the p53, the epidermal growth factor receptor (EGFr; c-*erb*B-1) and c-*erb*B-2 proteins was studied in 82 patients with primary transitional cell carcinoma of the bladder using an immuno-histochemical method. Strong or moderate staining was found in 18% of tumours for p53 with weaker staining in a further 36% giving a total of 54% of tumours stained for p53. Strong staining was found in 15% of tumours for c-*erb*B-2 and in 31% for the EGFr. Tumours invading the bladder muscle were significantly more likely to be strongly stained positively for p53 and/or EGFr compared with superficial tumours: only 15% of invasive tumours were stained negatively for both p53 and EGFr. No statistical association was found between p53 and EGFr expression. Weakly positive associations were found between the expression of c-*erb*B-2 and p53 and between muscle invasive tumours and increased expression of c-*erb*B-2. Alterations in the expression of p53, c-*erb*B-1 and c-*erb*B-2 were found frequently in human transitional cell carcinoma of the urinary bladder and may be of clinical use in defining patient sub-groups of differing prognosis.

p53 is a 393 amino-acid nucleo-phosphoprotein first identified as a result of binding to the large T antigen of the DNA virus, SV40 (Lane & Crawford, 1979). The p53 gene product binds strongly to the alpha subunit of DNA polymerase (Gannon & Lane, 1987), and E1B antigen of adenovirus and the E6 antigen of polyoma virus. It is a candidate gene for the control of cellular proliferation (Jenkins & Sturzbecher, 1988).The human gene is located on the short arm of chromosome 17 (Miller *et al.*, 1986) which is a common site for allelic deletions in human tumours, often being accompanied by mutations of the remaining allele (Baker *et al.*, 1989; Nigro *et al.*, 1989; Takahashi *et al.*, 1989; Thompson *et al.*, 1990). Transfection studies have shown that the wild type protein is able to suppress cell proliferation and transformation (Finlay *et al.*, 1989; Mercer *et al.*, 1990). However, cooperation has been demonstrated between mutant p53 and mutant *ras* genes in cellular transformation (Parada *et al.*, 1984; Hinds *et al.*, 1989) and transgenic mice carrying mutant p53 are at increased risk of malignancy (Lavigueur *et al.*, 1989). Initially, these data suggested that p53 may function as a tumour suppressor gene, but it now seems likely that mutations in p53 may also cause it to act as an oncogene under certain circumstances. The p53 protein can undergo self-oligomerisation (Kraiss *et al.*, 1988). It has been proposed that in cells with a single allelic mutation of p53, the mutated p53 which has a significantly increased half-life, oligomerises and inactivates the wild type protein. In addition, the mutated p53 product does not bind to large T antigen and unlike normal p53 (Braithwaite *et al.*, 1987) does not inhibit SV40 replication.

The c-*erb*B-2 gene encodes a membrane bound glycoprotein which has sequence similarity with the epidermal growth factor receptor (c-*erb*B-1 protein). Amplification and over-expression of this protein in carcinomas of the breast and ovary (Barnes, 1989; Slamon *et al.*, 1989) is associated with an inferior prognosis. The function of c-*erb*B-2 remains uncertain, although it is thought possibly to function as a growth factor receptor for an unidentified ligand.

Increased expression of the epidermal growth factor receptor (EGFr) is found in certain bladder (Neal *et al.*, 1985, 1990) and breast carcinomas (Sainsbury *et al.*, 1987; Lewis *et al.*, 1990). In breast carcinoma, a significant association has been found between p53 and the EGFr (Cattoretti *et al.*, 1988). In view of these findings and previous observations that increased expression of the EGFr is of potential prognostic significance in human bladder cancer (Neal *et al.*, 1990), we have carried out a study of p53, the EGFr and c-*erb*B-2 in human bladder cancer.

Correspondence: D.E. Neal, Department of Urology, Freeman Hospital, Freeman Road, Newcastle-upon-Tyne NE7 7DN, UK.
Received 8 November 1990; and in revised form 11 January 1991.

### Patients

Eighty-two patients (56 M; 26 F; mean age 69 ± 10 years) with newly diagnosed primary transitional cell carcinomas of the urinary bladder were studied. Tumour samples were taken by means of cystoscopic resection, the samples were placed in ice-cold saline and were urgently frozen and stored in liquid nitrogen. The tumours were staged by examination under anaesthesia (UICC, 1978) and according to the presence of invasion of lamina propria (pT1) or its absence (pTa) or invasion of detrusor muscle (T2-T4). In addition, paraffin embedded sections of each tumour were prepared and tumour grade was assessed.

Forty-eight patients had superficial tumours (pTa and pT1; 42 moderately differentiated tumours and six poorly differentiated tumours) and 33 had muscle invasive disease (six moderately differentiated; 27 poorly differentiated tumours). Tumour category was not assigned to one poorly differentiated tumour.

### Methods

Frozen sections were cut at 5 μ, air-dried and fixed in acetone for 10 min. The protein products of p53, the EGFr and c-*erb*B-2 were identified by means of an indirect immunoperoxidase technique using the following monoclonal antibodies. PAb240 recognises an evolutionarily conserved epitope on the p53 protein lying between amino acids 156–214 on murine p53 and it is highly specific for mutated protein as demonstrated by immuno-histochemistry, immuno-precipitation and immuno-blotting techniques (Gannon *et al.*, 1990). It does not bind normal p53 whereas many other monoclonal antibodies to p53 bind both normal and mutated p53. Sections were incubated at room temperature for 30 min with PAb240 as neat supernatant. NCL-CB11 (Novocastra Laboratories) was raised against a synthetic peptide from the C-terminal end of the predicted c-*erb*B-2 protein amino-acid sequence (Corbett *et al.*, 1990); culture medium diluted 1:40

was incubated with sections at 4°C overnight. EGFR1 (Amersham International plc) recognises an epitope on the external domain of the EGFr (Waterfield *et al.*, 1982) and was applied at a dilution of 1:40 for 30 min at room temperature. After incubation with primary antibody, sections were washed in tris-phosphate buffered saline (TBS) and covered with peroxidase-conjugated rabbit anti-mouse immuno-globulin (Dakopatts) diluted 1:20 for 30 min. The peroxidase reaction was developed using diaminobenzidine as chromogen and sections were counterstained with haematoxylin. In addition, 20 further sections were stained with monoclonal antibody PAb1801 (Oncogene Sciences) which binds to both mutated and normal p53 protein; it is specific for a denaturation resistant epitope between amino acids 32–79.

Sections of appropriate control material were included with each staining run: a squamous cell carcinoma of the bronchus known to show intense nuclear staining with PAb240, a breast carcinoma with approximately 15-fold amplification of the c-*erb*B-2 gene for NCL-CB11 and normal human skin and placenta for EGFR1. For each run, negative controls were prepared by staining duplicate sections for each tumour using the methods described above, but omitting the primary antibody. For occasional smaller tumours, insufficient tissue of suitable quantity was available to permit staining for p53 (three cases), EGFr (one case) or c-*erb*B-2 (one case).

Tumours were scored by assessing both the intensity of staining (on a four point scale: zero, weak, moderate, strong) and its extent (focal or diffuse). For the purpose of analysing the relationship between variables in the present study, tumours showing at least focal staining of moderate and strong staining intensity were taken as positive. Statistical analysis was performed by means of Chi square tests or Fisher's exact test.

### Results

### *Staining for p53*

Staining of strong or moderate intensity was found in 14 of 79 tumours (18%), weak staining was found in a further 29 cases (36%: total positive cases = 54%). Staining was identified in cell nuclei and had a diffuse, granular or clumped appearance, depending on the quality of the nuclear chromatin. No cases were seen of only cytoplasmic staining. There was no appreciable staining of stromal or inflammatory cells or non-dysplastic transitional cell urothelium. Twenty of the tumours (six strong or moderately positive; three weakly positive and 11 negative with PAb240) were studied with antibody PAb1801 which stains normal and mutated p53. There was a good correlation between the two antibodies: six of the 20 being strongly or moderately positive with PAb240 and seven of 20 being strongly or moderately positive with PAb1801. The 45% of tumours being positive with PAb240 remained positive, but three additional negative tumours stained giving an overall positive rate of 60% with PAb1801. The details were as follows: five of the six strongly or moderately positive tumours remained strongly or moderately positive and one was weakly positive on staining with PAb1801. Of the three weakly positive tumours, one became negative, one became moderately positive and one remained weakly positive on staining with PAb1801. Of the 11 negative tumours, one became moderately positive and two became weakly positive.

### *Staining for c-erbB-2 and the EGFr*

Previous studies have shown that both EGFR1 and NCL-CB11 may produce a combination of both cytoplasmic and membrane staining, but in the present study, only membrane staining was assessed. Strong or moderate staining with NCL-CB11 (regarded as positive for c-*erb*B-2) was observed in 12 of 81 tumours (15%) and weak staining observed in a further 19 tumours (23%). Strong or moderate staining with EGFR1 (regarded as positive for the EGFr) was found in 25 of 81 tumours (31%) and weak staining was observed in a further 43 tumours (53%).

### *Relationships among tumour stage, grade, p53, the EGFr and c-erbB-2*

Strong or moderate positive staining for p53 or the EGFr was associated significantly with muscle invasion and high tumour grade (poor differentiation; Tables I and II).

Complete data on tumour grade, stage and staining for p53, c-*erb*B-2 and the EGFr were available in 77 tumours. Combined staining for either p53 or the EGFr or both was strongly associated with stage and grade. Thirty-three of the 77 tumours invaded muscle and 28 of the 33 (85%) were stained positively for either p53 or the EGFr (Table III). On the other hand, only six of 44 superficial tumours (16%) were stained positively for either p53 or the EGFr (Table III). This difference was highly significant (Chi-square = 31.6; $P < 0.0001$). Of the 78 tumours in which data were available for p53 and the EGFr, positive staining for p53 and/or the EGFr was found in 28 of 34 (82%) poorly differentiated tumours, but only in seven of 44 moderately differentiated tumours (16%; Chi-square = 31.6; $P < 0.0001$).

On the other hand, the association between staining for c-*erb*B-2 and tumour stage was weak: eight of 32 invasive tumours (25%) and four of 48 superficial tumours were c-*erb*B-2 positive (8%; Fisher's exact test = 0.057). No correlation was found between c-*erb*B-2 and histological grade.

There was also a weak positive association between p53 positivity and positive staining for c-*erb*B-2. Five of 13 tumours (38%) which were positively stained for c-*erb*B-2 were also p53 positive compared with only nine of 68 c-*erb*B-2 negative tumours (13%; Fisher's exact test = 0.043). No evidence was found of co-expression of p53 protein and EGFr ($P = 0.99$) or EGFr and c-*erb*B-2 protein ($P = 0.75$).

### Discussion

Mutation of the p53 gene appears to be the commonest mutation yet found – being identified in about 50% of a variety of human tumours including breast (Cattoretti *et al.*, 1988), colon (van den Berg *et al.*, 1989) and lung (Iggo *et al.*, 1990). The present study provides evidence of strong staining

**Table I** Correlation of tumour stage with strong or moderate staining for p53 or EGFr

| | *p53* | | *EGFr* | |
|---|---|---|---|---|
| | *– ve* | *+ ve* | *– ve* | *+ ve* |
| Superficial tumours (pTa, pT1) | 44 | 1 | 42 | 5 |
| Invasive tumours (T2, T3, T4) | 21 | 12 | 13 | 20 |

Chi-square = 13.6 ($P < 0.001$); Chi-square = 20.3 $P < 0.001$)

**Table II** Correlation of differentiation of the tumour with strong or moderate staining for p53 and EGFr

| | *p53* | | *EGFr* | |
|---|---|---|---|---|
| | *– ve* | *+ ve* | *– ve* | *+ ve* |
| Well + moderate | 43 | 2 | 42 | 5 |
| Poor | 22 | 12 | 14 | 20 |

Chi-square = 10.6 ($P < 0.005$); Chi-square = 19.3 ($P < 0.001$)

**Table III** Relationship between strong and moderate staining for p53 and the EGFr in 33 muscle invasive and 44 superficial tumours

| | *Invasive (n = 33)* *p53* | | | *Superficial (n = 44)* *p53* | |
|---|---|---|---|---|---|
| | *– ve* | *+ ve* | | *– ve* | *+ ve* |
| EGFr – ve | 5 | 8 | EGFR – ve | 38 | 1 |
| EGFr + ve | 16 | 4 | EGFR + ve | 5 | 0 |

in 18% of primary human transitional cell carcinomas of the bladder with weaker staining found in a further 36%.

Allelic deletion of chromosome 17 p has been demonstrated in human tumours, including bladder cancers (Yokota *et al.*, 1987; Vogelstein *et al.*, 1988; Mackay *et al.*, 1988; Tsai *et al.*, 1990) and such deletion is frequently accompanied by mutation of the remaining allele (Baker *et al.*, 1989; Nigro *et al.*, 1989; Takahasi *et al.*, 1989). Iggo and colleagues found single point mutations in p53 messenger RNA in each of three lung cancers showing immuno-histochemical evidence of increased p53 protein expression (Iggo *et al.*, 1990). This evidence is consistent with previous observations that mutation of the p53 protein is usually associated with an increased half-life in rodents (Jenkins & Sturzbecher, 1988). A large number of different point mutations have been observed in the p53 gene (Nigro *et al.*, 1989) and the monoclonal antibody PAb240 identifies many, but not all, of these mutations (Bartek *et al.*, 1990; Rodrigues *et al.*, 1990). It is therefore likely that some mutated copies of p53 were not detected by our study. There is good evidence that positive staining with PAb240 is a specific sign of mutated p53 as this antibody binds to a variety of mutants in which there is a common conformational change in the protein (Gannon *et al.*, 1990; Bartek *et al.*, 1990; Rodrigues *et al.*, 1990). However, there is also evidence that certain mutants do not carry an exposed PAb240 epitope (Rodrigues *et al.*, 1990).

It seems likely that similar mechanisms underly our present observations in bladder cancer. Determination of the exact incidence of point mutations of the p53 gene in bladder cancer will probably involve sequencing techniques. Eighteen per cent of bladder tumours expressed p53 protein strongly in the present study, but a further 36% exhibited weakly positive staining giving a total of 54% positively stained. Further molecular biological studies will be necessary to clarify the significance of this weak positive staining, but it is likely that these weakly positive tumours also contain mutated p53, as variation in staining intensity has been noted previously even in cells known to contain mutated p53. However, from recent studies of colo-rectal cancer, it is also likely that a further number of the negatively stained tumours will contain mutated p53 protein in which the PAb240 epitope has not been exposed (Rodrigues *et al.*, 1990). Interestingly, we found a significant association between strong or moderate staining and muscle invasion suggesting that tumours with a high risk of progression may contain high levels of mutated p53 protein. One may speculate from the clinical point of view that such high intensities of staining may provide useful prognostic information.

It is possible that other antibodies which bind to different epitopes of the p53 protein may prove useful in identifying the mutations in p53 which do not produce the conformational changes identified by PAb240. However, previous studies using PAb1801 and PAb240 have shown similar patterns of staining (Bartek *et al.*, 1990; Rodrigues *et al.*, 1990). In the present study, we also found broadly similar patterns of staining – a similar number being strongly or moderately positive with each antibody, although PAb1801 stained a few more tumours weakly positive.

Determination of quantitative or qualitative changes in the expression of oncogenes and their protein products might prove of clinical use in classifying tumours into different prognostic categories given that such changes at the cellular level may lead directly to alterations in tumour behaviour. Such information might well supplement traditional prognostic factors such as tumour grade and stage – indeed this has been shown to be the case in bladder cancer for the EGFr (Neal *et al.*, 1990) and in breast cancer for the EGFr and c-*erb*B-2 (Sainsbury *et al.*, 1987; Wright *et al.*, 1989). No correlation was found in the present study between expression of p53 and the EGFr in contrast to a previous study of breast cancer (Cattoretti *et al.*, 1988) which used a different antibody against p53. On the other hand, 85% of the muscle invasive cancers in our study were stained positively for the EGFr and/or p53 and the muscle invasive group included all four double positive tumours. Obviously, it will be of interest to follow-up this cohort of patients to determine future clinical behaviour, particularly in the few patients with invasive disease who are negative for both p53 and the EGFr and with superficial tumours which are positive for p53 or the EGFr.

In an initial investigation of 44 patients, positive staining (of all intensities) for the c-*erb*B-2 protein product was observed in 36% (Wright *et al.*, 1990), although a previous study found little evidence of increased c-*erb*B-2 expression using paraffin embedded material and a different antibody (McCann *et al.*, 1990). The mechanisms underlying overexpression of c-*erb*B-2 in bladder cancer are unclear, but in cancers of the breast and ovary it is associated with gene amplification (Venter *et al.*, 1987; Gusterson *et al.*, 1988; Slamon *et al.*, 1989), and a poor clinical outcome. The weak correlation found between expression of c-*erb*B-2 and tumour stage in the present study of bladder cancer does not exclude a potential role as a prognostic factor, for in breast cancer also, no consistent correlation has been found despite a strong association between c-*erb*B-2 expression and poor survival (Barnes, 1989).

This study was supported by a grant from the North of England Cancer Research Campaign.

### References

BAKER, S.J., FEARON, E.R., NIGRO, J.M. & 9 others (1989). Chromosome 17 deletions and p53 gene mutations in colorectal carcinomas. *Science*, **244**, 217.

BARNES, D.M. (1989). Breast cancer and a proto-oncogene. *Br. Med. J.*, **299**, 1061.

BARTEK, J., IGGO, R., GANNON, J. & LANE, D.P. (1990). Genetic and immunochemical analysis of mutant p53 in human breast cancer. *Oncogene*, **5**, 893.

BRAITHWAITE, A.W., STURZBECHER, H.-W., ADDISON, C., PALMER, C., RUDGE, K. & JENKINS, J.R. (1987). Mouse p53 inhibits SV40 origin-dependent DNA replication. *Nature*, **329**, 458.

CATTORETTI, G., RILKE, F., ANDREOLA, S., D'AMATO, L. & DELIA, D. (1988). p53 expression in breast cancer. *Int. J. Cancer*, **41**, 178.

CORBETT, I.P., HENRY, J.A., ANGUS, B. & 8 others (1990). NCL-CB11, a new monoclonal antibody recognising the internal domain of the c-*erb*B-2 oncogene protein effective for use on formalin-fixed, paraffin embedded tissue. *J. Pathol.*, **161**, 15.

FINLAY, C.A., HINDS, P.W. & LEVINE, A.J. (1989). The p53 proto-oncogene can act as a suppressor of transformation. *Cell*, **57**, 1083.

GANNON, J.V. & LANE, D.P. (1987). p53 and DNA polymerase-a compete for binding to SV40 T antigen. *Nature*, **329**, 456.

GANNON, J.V., GREAVES, R. & LANE, D.P. (1990). Activating mutations in p53 produce a common conformational effect. A monoclonal antibody specific for the mutant form. *EMBO J.*, **9**, 1595.

GUSTERSON, B.A., GULLICK, W.J., VENTNER, D.J. & 5 others (1988). Immunohistochemical localisation of c-*erb*B-2 in human breast carcinomas. *Mol. Cell. Probes.* **2**, 383.

HINDS, P., FINLAY, C. & LEVINE, A.J. (1989). Mutation is required to activate the p53 gene for cooperation with the *ras* oncogene and its transformation. *J. Virol.*, **63**, 739.

IGGO, R., GATTER, K., BARTEK, J., LANE, D. & HARRIS, A.L. (1990). Increased expression of mutant forms of p53 oncogene in primary lung cancer. *Lancet*, **i**, 675.

JENKINS, J.R. & STURZBECHER, H.-W. (1988). The p53 oncogene. In: *The Oncogene Handbook*. Reddy E.P. (ed.). Elsevier: Amsterdam, pp. 403–423.

KRAISS, S., QUASIER, A., OREN, M. & MONTENARH, M. (1988). Oligomerisation of oncoprotein p53. *J. Virol.*, **62**, 4737.

LANE, D.P. & CRAWFORD, L.V. (1979). T antigen is bound to a host protein in SV40 transformed cells. *Nature*, **278**, 261.

LAVIGUEUR, A., MALTBY, V., MOCK, D., ROSSANT, J., PAWSON, T. & BERNSTEIN, A. (1989). High incidence of lung, bone and lymphoid tumors in transgenic mice overexpressing mutant alleles of the p53 oncogene. *Mol. Cell. Biol.*, **9**, 3982.

LEWIS, S., LOCKER, A., TODD, J.H. & 5 others (1990). Expression of epidermal growth factor receptor in breast carcinoma. *J. Clin. Pathol.*, **43**, 385.

McCANN, A., DERVAN, P.A., JOHNSTON, P.A., GULLICK, W.J. & CARNEY, D.N. (1990). c-*erb*B-2 oncoprotein expression in primary human tumours. *Cancer*, **65**, 88.

MACKAY, J., ELDER, P.A., STEEL, C.M., FORREST, A.P.M. & EVANS, H.J. (1988). Allele loss on short arm of chromosome 17 in breast cancer. *Lancet*, **ii**, 1384.

MERCER, W.E., AMIN, M., SAUVE, G.J., APELLA, E., ULLRICH, S.J. & ROMANO, J.W. (1990). Wild type human p53 is antiproliferative in SV40-transformed hamster cells. *Oncogene*, **5**, 973.

MILLER, C., MOHANDAS, T., WOLF, D., PROKOCIMER, M., ROTTER, V. & KOEFFLER, H.P. (1986). Human p53 gene is localised to short arm of chromosome 17. *Nature*, **319**, 783.

NEAL, D.E., MARSH, C., BENNETT, M.K. & 4 others (1985). Epidermal growth factor receptors in human bladder cancer: comparison of invasive and superficial tumours. *Lancet*, **i**, 366.

NEAL, D.E., SHARPLES, L., SMITH, K., FENNELLY, J.A., HALL, R.R. & HARRIS, A.L. (1990). The epidermal growth factor receptor and the prognosis of bladder cancer. *Cancer*, **65**, 1619.

NIGRO, J.M., BAKER, S.J. & PREISINGER, A.C. (1989). Mutations in the p53 gene occur in diverse tumour types. *Nature*, **342**, 705.

PARADA, L.F., LAND, H., WEINBERG, R.A., WOLF, D. & ROTTER, V. (1984). Cooperation between gene encoding p53 tumor antigen and ras in cellular transformation. *Nature*, **312**, 649.

RODRIGUES, N.R., ROWAN, A., SMITH, M.E.F. & 4 others (1990). p53 mutations in colorectal cancer. *Proc. Natl Acad. Sci. USA*, **87**, 7555.

SAINSBURY, J.R.C., FARNDON, J.R., NEEDHAM, G.K., MALCOLM, A.J. & HARRIS, A.L. (1987). Epidermal growth factor receptor status as predictor of early recurrence of and death from breast cancer. *Lancet*, **i**, 1398.

SLAMON, D.J., GODOLPHIN, W., JONES, L.A. & 8 others (1989). Studies of the HER/neu proto-oncogene in human breast and ovarian cancer. *Science*, **244**, 707.

TAKAHASHI, T., NAU, M.M., CHIBA, I. & 7 others (1989). p53: a frequent target for genetic abnormalities in lung cancer. *Science*, **246**, 491.

THOMPSON, A.M., STEEL, C.M., CHETTY, U. & 5 others (1990). p53 gene mRNA expression and chromosome 17p allele loss in breast cancer. *Br. J. Cancer*, **61**, 74.

TSAI, Y.C., NICHOLS, P.W., HITI, A.L., WILLIAMS, Z., SKINNER, D.G. & JONES, P.A. (1990). Allelic loss of chromosomes 9, 11, and 17 in human bladder cancer. *Cancer Res.*, **50**, 44.

UICC (UNION INTERNATIONALE CONTRE LE CANCER). (1978). *TNM Classification of Malignant Tumours*. Third Edition, Geneva: International Union Against Cancer.

VAN DEN BERG, F.M., TIGGES, A.J., SCHIPPER, M.E.I., DEN HARTOG-JEGER, F.C.A., KROES, W.G.M. & WALBLOOMERS, J.M.M. (1989). Expression of the nuclear oncogene p53 in colon tumors. *J. Pathol.*, **157**, 193.

VENTER, D.J., TUZI, N.L., KUKAR, S. & GULLICK, W.J. (1987). Over expression of the c-*erb*B-2 oncoprotein in breast cancer: immunohistochemical assessment correlates with gene amplification. *Lancet*, **ii**, 69.

VOGELSTEIN, B., FEARON, E.R., HAMILTON, S.R. & 7 others (1988). Genetic alterations during colorectal tumor development. *N. Engl. J. Med.*, **319**, 525.

WATERFIELD, M.D., SCRACE, G.T., WHITTLE, N. & 5 others (1982). A monoclonal antibody to the human epidermal growth factor receptor. *J. Cell. Biochem.*, **20**, 149.

WRIGHT, C., ANGUS, B., NICHOLSON, S. & 6 others (1989). Expression of c-*erb*B-2 oncoprotein: a prognostic indicator in human breast cancer. *Cancer Res.*, **49**, 2087.

WRIGHT, C., MELLON, K., NEAL, D.E., JOHNSON, P., CORBETT, I.P. & HORNE, C.H.W. (1990). Expression of c-*erb*B-2 (neu) protein product in bladder cancer. *Br. J. Cancer*, **62**, 764.

YOKOTA, J., WADA, M., SHIMOSATO, Y., TERADA, M. & SUGIMURA, T. (1987). Loss of heterozygosity on chromosome 3, 13 and 17 in small cell carcinoma and on chromosome 3 in adenocarcinoma of the lung. *Proc. Natl Acad. Sci. USA*, **84**, 9252.


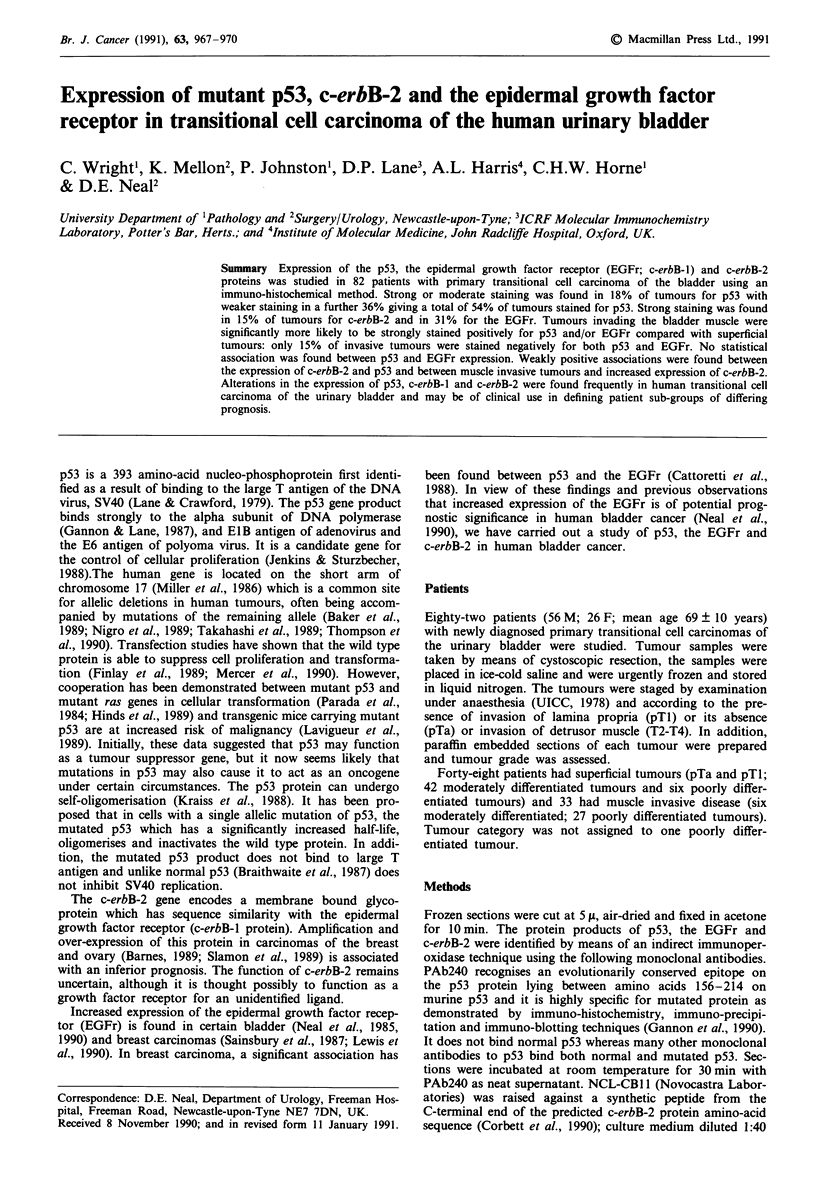

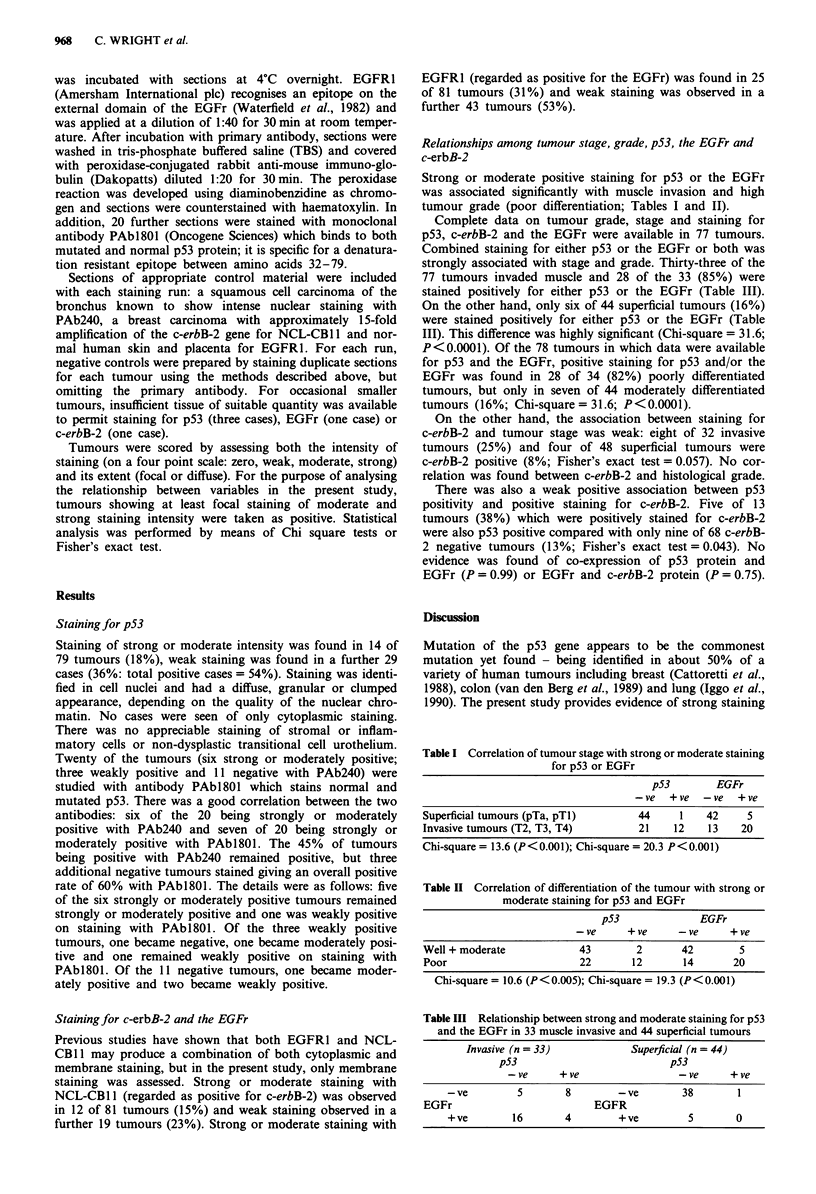

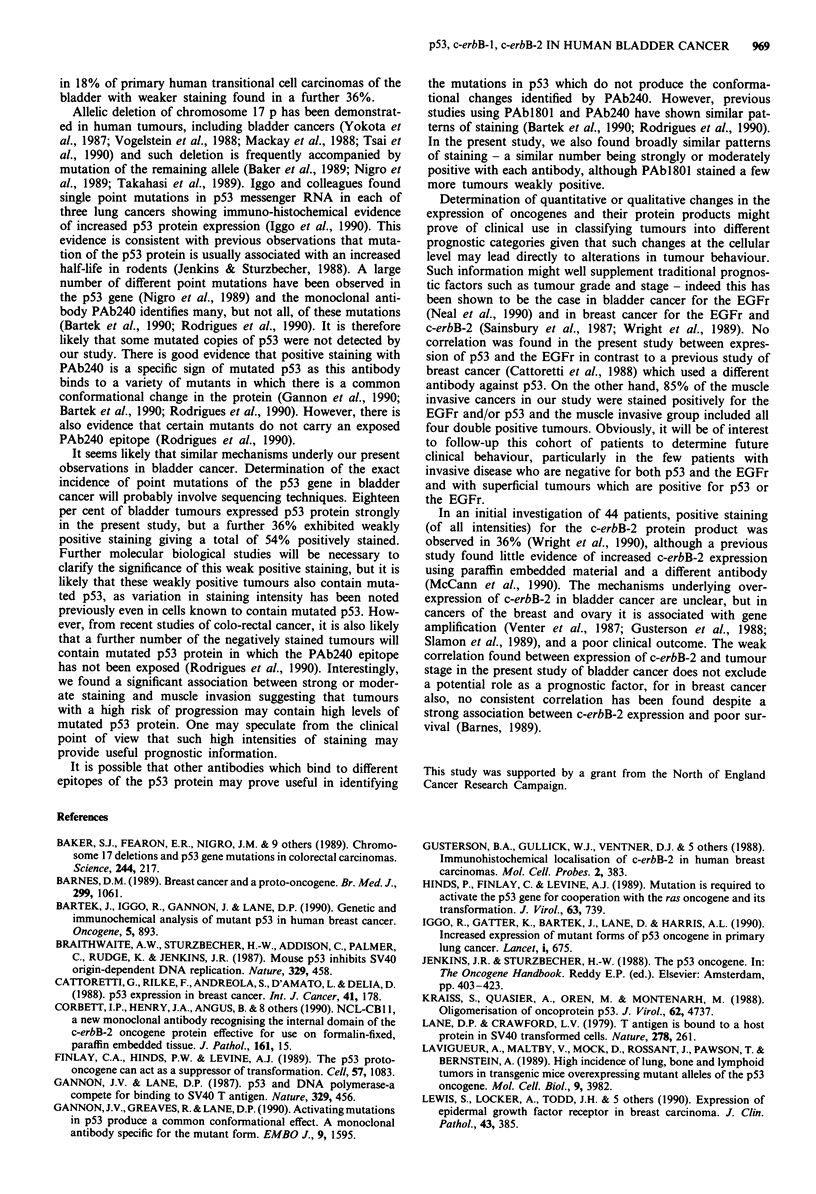

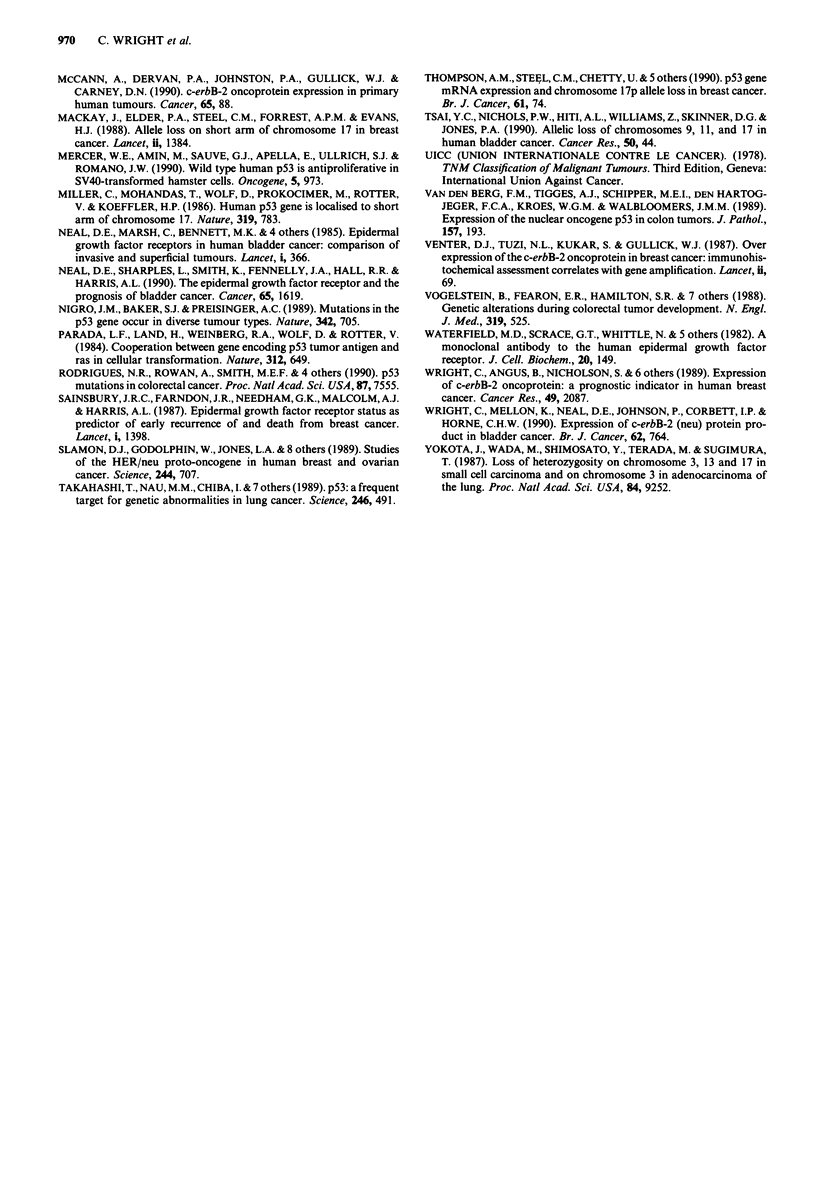


## References

[OCR_00402] Baker S. J., Fearon E. R., Nigro J. M., Hamilton S. R., Preisinger A. C., Jessup J. M., vanTuinen P., Ledbetter D. H., Barker D. F., Nakamura Y. (1989). Chromosome 17 deletions and p53 gene mutations in colorectal carcinomas.. Science.

[OCR_00407] Barnes D. M. (1989). Breast cancer and a proto-oncogene.. BMJ.

[OCR_00411] Bartek J., Iggo R., Gannon J., Lane D. P. (1990). Genetic and immunochemical analysis of mutant p53 in human breast cancer cell lines.. Oncogene.

[OCR_00416] Braithwaite A. W., Sturzbecher H. W., Addison C., Palmer C., Rudge K., Jenkins J. R. (1987). Mouse p53 inhibits SV40 origin-dependent DNA replication.. Nature.

[OCR_00421] Cattoretti G., Rilke F., Andreola S., D'Amato L., Delia D. (1988). P53 expression in breast cancer.. Int J Cancer.

[OCR_00431] Finlay C. A., Hinds P. W., Levine A. J. (1989). The p53 proto-oncogene can act as a suppressor of transformation.. Cell.

[OCR_00438] Gannon J. V., Greaves R., Iggo R., Lane D. P. (1990). Activating mutations in p53 produce a common conformational effect. A monoclonal antibody specific for the mutant form.. EMBO J.

[OCR_00434] Gannon J. V., Lane D. P. (1987). p53 and DNA polymerase alpha compete for binding to SV40 T antigen.. Nature.

[OCR_00448] Hinds P., Finlay C., Levine A. J. (1989). Mutation is required to activate the p53 gene for cooperation with the ras oncogene and transformation.. J Virol.

[OCR_00453] Iggo R., Gatter K., Bartek J., Lane D., Harris A. L. (1990). Increased expression of mutant forms of p53 oncogene in primary lung cancer.. Lancet.

[OCR_00463] Kraiss S., Quaiser A., Oren M., Montenarh M. (1988). Oligomerization of oncoprotein p53.. J Virol.

[OCR_00467] Lane D. P., Crawford L. V. (1979). T antigen is bound to a host protein in SV40-transformed cells.. Nature.

[OCR_00471] Lavigueur A., Maltby V., Mock D., Rossant J., Pawson T., Bernstein A. (1989). High incidence of lung, bone, and lymphoid tumors in transgenic mice overexpressing mutant alleles of the p53 oncogene.. Mol Cell Biol.

[OCR_00477] Lewis S., Locker A., Todd J. H., Bell J. A., Nicholson R., Elston C. W., Blamey R. W., Ellis I. O. (1990). Expression of epidermal growth factor receptor in breast carcinoma.. J Clin Pathol.

[OCR_00489] Mackay J., Steel C. M., Elder P. A., Forrest A. P., Evans H. J. (1988). Allele loss on short arm of chromosome 17 in breast cancers.. Lancet.

[OCR_00494] Mercer W. E., Amin M., Sauve G. J., Appella E., Ullrich S. J., Romano J. W. (1990). Wild type human p53 is antiproliferative in SV40-transformed hamster cells.. Oncogene.

[OCR_00499] Miller C., Mohandas T., Wolf D., Prokocimer M., Rotter V., Koeffler H. P. Human p53 gene localized to short arm of chromosome 17.. Nature.

[OCR_00504] Neal D. E., Marsh C., Bennett M. K., Abel P. D., Hall R. R., Sainsbury J. R., Harris A. L. (1985). Epidermal-growth-factor receptors in human bladder cancer: comparison of invasive and superficial tumours.. Lancet.

[OCR_00509] Neal D. E., Sharples L., Smith K., Fennelly J., Hall R. R., Harris A. L. (1990). The epidermal growth factor receptor and the prognosis of bladder cancer.. Cancer.

[OCR_00514] Nigro J. M., Baker S. J., Preisinger A. C., Jessup J. M., Hostetter R., Cleary K., Bigner S. H., Davidson N., Baylin S., Devilee P. (1989). Mutations in the p53 gene occur in diverse human tumour types.. Nature.

[OCR_00518] Parada L. F., Land H., Weinberg R. A., Wolf D., Rotter V. (1984). Cooperation between gene encoding p53 tumour antigen and ras in cellular transformation.. Nature.

[OCR_00523] Rodrigues N. R., Rowan A., Smith M. E., Kerr I. B., Bodmer W. F., Gannon J. V., Lane D. P. (1990). p53 mutations in colorectal cancer.. Proc Natl Acad Sci U S A.

[OCR_00526] Sainsbury J. R., Farndon J. R., Needham G. K., Malcolm A. J., Harris A. L. (1987). Epidermal-growth-factor receptor status as predictor of early recurrence of and death from breast cancer.. Lancet.

[OCR_00532] Slamon D. J., Godolphin W., Jones L. A., Holt J. A., Wong S. G., Keith D. E., Levin W. J., Stuart S. G., Udove J., Ullrich A. (1989). Studies of the HER-2/neu proto-oncogene in human breast and ovarian cancer.. Science.

[OCR_00537] Takahashi T., Nau M. M., Chiba I., Birrer M. J., Rosenberg R. K., Vinocour M., Levitt M., Pass H., Gazdar A. F., Minna J. D. (1989). p53: a frequent target for genetic abnormalities in lung cancer.. Science.

[OCR_00546] Tsai Y. C., Nichols P. W., Hiti A. L., Williams Z., Skinner D. G., Jones P. A. (1990). Allelic losses of chromosomes 9, 11, and 17 in human bladder cancer.. Cancer Res.

[OCR_00562] Venter D. J., Tuzi N. L., Kumar S., Gullick W. J. (1987). Overexpression of the c-erbB-2 oncoprotein in human breast carcinomas: immunohistological assessment correlates with gene amplification.. Lancet.

[OCR_00568] Vogelstein B., Fearon E. R., Hamilton S. R., Kern S. E., Preisinger A. C., Leppert M., Nakamura Y., White R., Smits A. M., Bos J. L. (1988). Genetic alterations during colorectal-tumor development.. N Engl J Med.

[OCR_00578] Wright C., Angus B., Nicholson S., Sainsbury J. R., Cairns J., Gullick W. J., Kelly P., Harris A. L., Horne C. H. (1989). Expression of c-erbB-2 oncoprotein: a prognostic indicator in human breast cancer.. Cancer Res.

[OCR_00583] Wright C., Mellon K., Neal D. E., Johnston P., Corbett I. P., Horne C. H. (1990). Expression of c-erbB-2 protein product in bladder cancer.. Br J Cancer.

[OCR_00588] Yokota J., Wada M., Shimosato Y., Terada M., Sugimura T. (1987). Loss of heterozygosity on chromosomes 3, 13, and 17 in small-cell carcinoma and on chromosome 3 in adenocarcinoma of the lung.. Proc Natl Acad Sci U S A.

[OCR_00558] van den Berg F. M., Tigges A. J., Schipper M. E., den Hartog-Jager F. C., Kroes W. G., Walboomers J. M. (1989). Expression of the nuclear oncogene p53 in colon tumours.. J Pathol.

